# Female Sexual Function After Radical Treatment for MIBC: A Systematic Review

**DOI:** 10.3390/jpm15090415

**Published:** 2025-09-02

**Authors:** Francesco Pio Bizzarri, Marco Campetella, Salvatore Marco Recupero, Fabrizio Bellavia, Lorenzo D’Amico, Francesco Rossi, Filippo Gavi, Giovanni Battista Filomena, Pierluigi Russo, Giuseppe Palermo, Nazario Foschi, Angelo Totaro, Mauro Ragonese, Maria Chiara Sighinolfi, Marco Racioppi, Emilio Sacco, Bernardo Rocco

**Affiliations:** 1 Department of Urology, Ospedale Isola Tiberina-Gemelli Isola, 00186 Rome, Italy; marco.campetella.fw@fbf-isola.it (M.C.); salvatoremarco.recupero@fbf-isola.it (S.M.R.); lorenzo.damico01@icatt.it (L.D.); emilio.sacco@unicatt.it (E.S.); 2 Department of Urology, Fondazione Policlinico Universitario Agostino Gemelli, 00168 Rome, Italy; fabrizio.bellavia01@icatt.it (F.B.); francesco.rossi04@icatt.it (F.R.); filippo.gavi01@icatt.it (F.G.); giovanni.filomena01@icatt.it (G.B.F.); giuseppe.palermo@policlinicogemelli.it (G.P.); nazario.foschi@policlinicogemelli.it (N.F.); angelo.totaro@policlinicogemelli.it (A.T.); mauro.ragonese@policlinicogemelli.it (M.R.); mariachiara.sighinolfi@policlinicogemelli.it (M.C.S.); marco.racioppi@policlinicogemelli.it (M.R.); bernardo.rocco@policlinicogemelli.it (B.R.); 3 Department of Life Science, Health, and Health Professions, University Unilink, 00165 Rome, Italy

**Keywords:** bladder cancer, radical cystectomy, radiotherapy, female sexual dysfunction

## Abstract

**Background:** Sexuality in women with muscle-invasive bladder cancer (MIBC) undergoing radical treatment represents a crucial aspect of their overall quality of life, which is increasingly recognized as a key component of patient-centered care and long-term well-being. This review aimed to analyze the available literature to provide a comprehensive overview of the effects of treatments on female sexual function. **Methods:** We included all qualitative and quantitative studies addressing sexual function in patients treated for MIBC. Excluded were narrative reviews, case reports, conference abstracts, systematic reviews, and meta-analyses. The included studies involved women undergoing either robot-assisted radical cystectomy (RARC) or open RC (ORC), often with nerve-sparing, vaginal-sparing, or pelvic organ-preserving techniques. Data on oncological and functional outcomes were collected. **Results:** A systematic review of 29 studies including 1755 women was conducted. RC was performed via robotic/laparoscopic approaches in 39% of cases and open techniques in 61%. Urinary diversions included orthotopic neobladders (48%), ileal conduits (42%), ureterocutaneostomies (3%), and Indiana pouches (7%). Radiotherapy, used in 6% of patients, was mainly applied in a curative, trimodal setting. Sexual function was evaluated using various pre- and/or postoperative questionnaires, most commonly the EORTC QLQ-C22, FACT-BL, Bladder Cancer Index (BCI), LENT SOMA, and Female Sexual Function Index (FSFI). Radiotherapy was associated with reduced sexual function, though outcomes were somewhat better than with surgery. Among surgical approaches, no differences in sexual outcomes were observed. **Conclusions:** Further qualitative research is essential to better understand the experience of FSD after treatment. Incorporating both patient and clinician perspectives will be key to developing tailored interventions. In addition, efforts should be made to standardize the questionnaires used to assess female sexual dysfunction, in order to improve comparability across studies and ensure consistent evaluation.

## 1. Introduction

Bladder cancer (BC) is a significant global health concern, ranking among the top 10 most common cancers worldwide. While men are more frequently affected, women diagnosed with BC often experience more aggressive disease, leading to a higher proportion requiring radical treatment [1,2]

Treatment for both muscle-invasive (MIBC) and non-muscle-invasive BC (NMIBC) can have substantial acute and long-term consequences [3]. Although research has focused on disease control, long-term effects, particularly for women, remain understudied. Existing evidence primarily examines male experiences, especially regarding sexual function.

Radical cystectomy (RC), representing the primary treatment for MIBC or recurrent high-risk NMIBC, often involves extensive surgery in women, including removal of the bladder, uterus, ovaries, and anterior vaginal wall [4]. Modern surgical approaches aim to preserve clitoral function and potentially the vagina and uterus, with a greater emphasis on maintaining female sexual function [5,6].

The advent of robotic surgery and the development of multiple platforms have made one of the surgeries with the highest rates of peri- and postoperative comorbidities more manageable for patients. Additionally, minimally invasive laparoscopic surgery allows for precise surgical procedures, enabling the preservation of nerves, blood supply, and, in women, reproductive organs [7,8].

For patients with muscle-invasive bladder cancer, radical radiotherapy can be an alternative to RC, particularly for those who desire organ preservation. Growing evidence supports its effectiveness in controlling the disease and reducing treatment side effects. However, lifelong endoscopic surveillance is essential in these cases [9].

Traditionally, RC in women involved the removal of the bladder, uterus, ovaries, fallopian tubes, and other pelvic structures. However, with the increasing use of ON reconstruction and the focus on improving postoperative quality of life (QoL), surgeons are exploring less radical approaches [10,11]. Since direct involvement of female reproductive organs in bladder cancer is relatively uncommon, organ-sparing cystectomy, which preserves the uterus, ovaries, and other structures, has become an option in select cases [12].

Careful patient selection is crucial to ensure that cancer control is not compromised. Factors considered include age, menopausal status, sexual function, fertility goals, and gynecologic history. Women with good overall health, younger age (though not a strict requirement), and a desire for organ preservation and neobladder reconstruction are generally considered better candidates for organ-sparing cystectomy [13].

FSD is a multifaceted condition with both physical and psychological origins. It affects an estimated 41% of premenopausal women globally. Age and underlying health conditions are known risk factors. FSD can manifest in various ways, including difficulties with arousal, orgasm, or pain. These issues can negatively impact body image, self-esteem, and intimate relationships, significantly affecting the quality of life for both the patient and her partner [14].

Both functional and oncologic outcomes are crucial in ensuring a good QoL for these patients and are, therefore, integral to the success of their treatment. The aim of this systematic review is to analyze the impact of radical treatment in patients with MIBC, with the goal of providing a comprehensive overview and informing future treatment decisions.

## 2. Material and Methods

This systematic review was conducted in accordance with the Preferred Reporting Items for Systematic Reviews and Meta-Analyses (PRISMA) guidelines (Supplementary Materials).

### 2.1. Search Methods

The research strategy involved collecting data from 3 databases: PubMed, Cochrane, and Scopus; we collected all articles within a time frame from 2000 to 2023. The search was conducted using Boolean operators and keywords as follows: for RC, we used ‘bladder cancer’ OR ‘urothelial carcinoma’ OR ‘urinary bladder neoplasm’ AND ‘radical cystectomy’ OR ‘cystectomy’ AND ‘women’ OR ‘female’ AND ‘sexual function’ OR ‘functional outcome’ OR ‘quality of life’ OR ‘QOL’, while for RT, we used ‘bladder cancer’ OR ‘urothelial carcinoma’ OR ‘urinary bladder neoplasm’ AND ‘radiation’ OR ‘radiotherapy’ OR ‘radiation therapy ‘OR ‘TMT’ OR ‘trimodal therapy’ AND ‘women’ OR ‘female’ AND ‘sexual function’ OR ‘functional outcome’ OR ‘quality of life’ OR ‘QOL’. This systematic review was prospectively registered in the PROSPERO international database of systematic reviews (registration number: CRD420251047362).

### 2.2. Inclusion Criteria

All qualitative and quantitative studies related to sexual function after RC or RT were included. Studies analyzing sexuality-related quality of life in women were included, while studies reporting sexual function data without distinguishing between male and female subjects were excluded.

### 2.3. Exclusion Criteria

We excluded all studies not in the English language, narrative reviews, systematic reviews, meta-analyses, and case reports.

### 2.4. Screening Procedure

The results were screened by two reviewers, initially by reading the title and abstract, and then by reading the full article. Expert opinions and conference abstracts were excluded.

**Figure 1:**
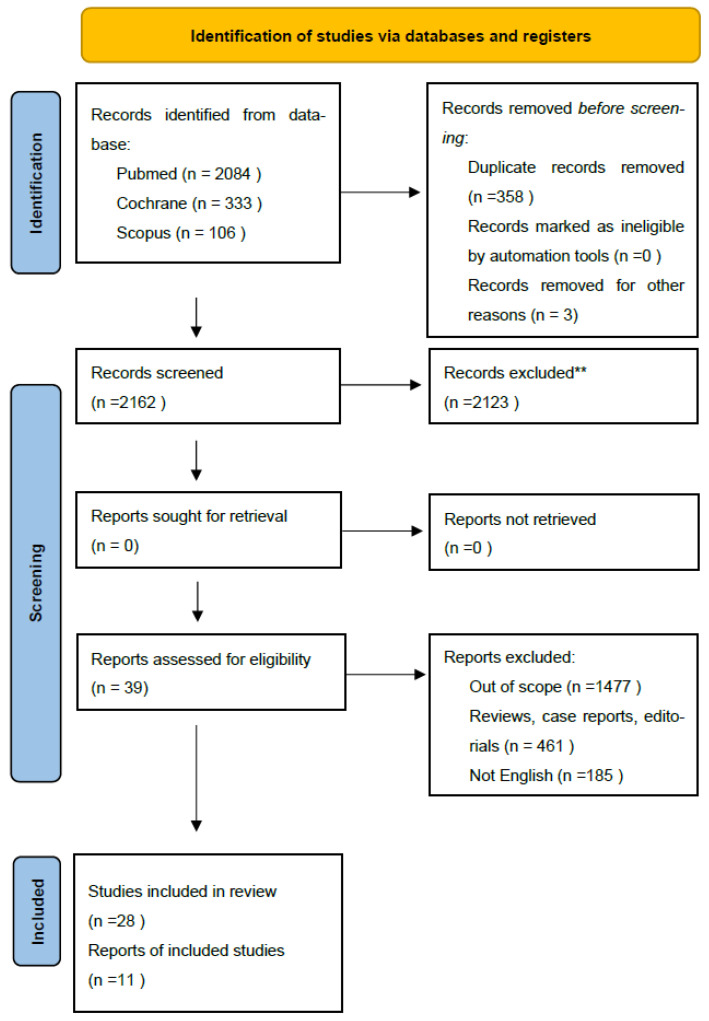
PRISMA 2020 flow diagram for selected studies Search captured all articles from Scopus, Pubmed, Cochrane. ** (refer to exclusion criteria).

To ensure comprehensive coverage, we manually reviewed the reference lists of all included studies for additional relevant articles. Figure 1 provides a flowchart illustrating the study screening process. All included studies underwent rigorous quality assessment using the appropriate Critical Appraisal Skills Programme (CASP) tool. Studies were excluded from the review if they failed to meet two or more of the predefined CASP quality criteria.

Data extracted from each full-text article included: study type, disease grade and stage, mean patient age, details on pre- and post-treatment counseling, treatment modality, patient-reported outcome measures employed, prevalence and types of sexual dysfunction (including sexual interest, enjoyment, intimacy concerns, and distress), identification of common themes, and reporting of female-specific data. Two independent reviewers conducted data extraction, with one reviewer performing the initial extraction and the other conducting a thorough check. Discrepancies in extracted data or quality assessments were resolved through discussion and consensus between the reviewers (Figure 1).

### 2.5. Risk of Bias

**Figure 2:**
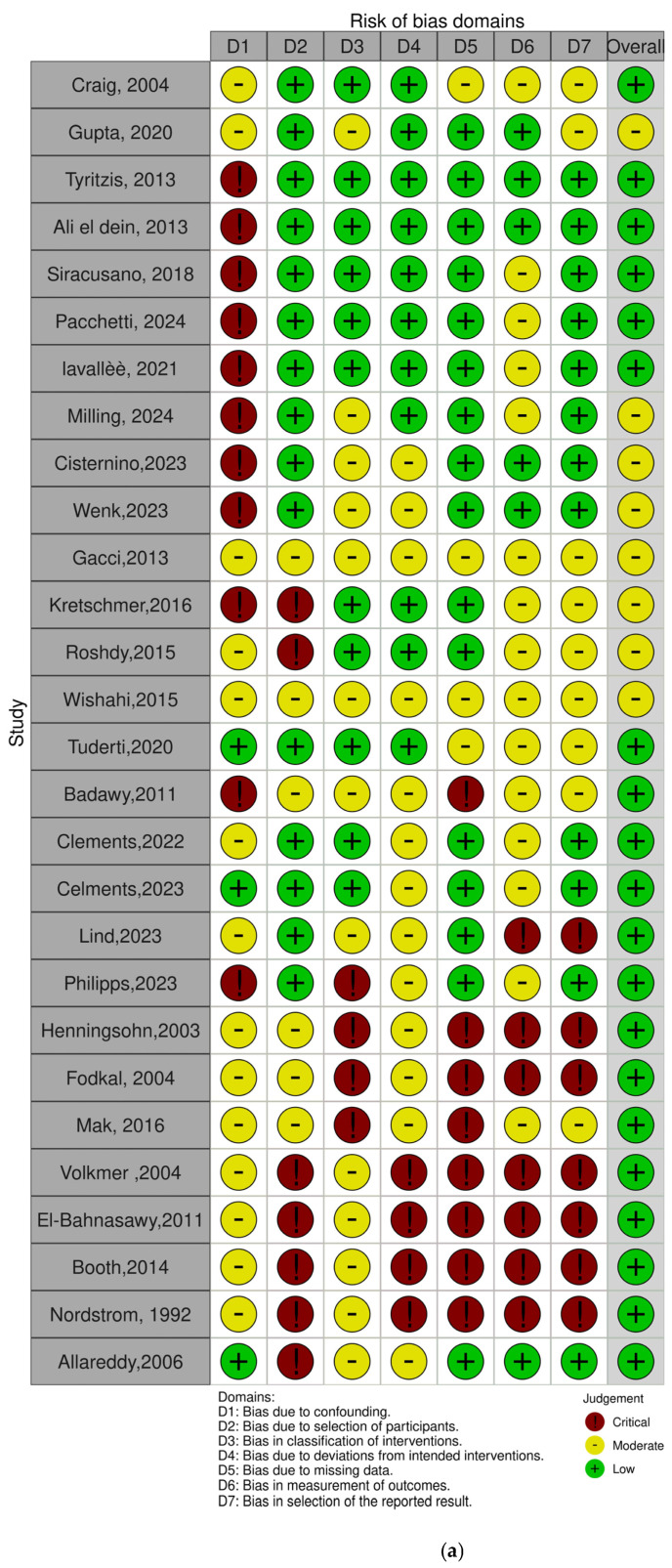

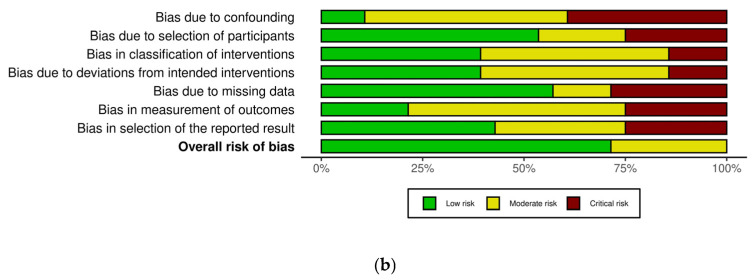
(**a**,**b**). Assessment for risk of bias with ROBINS-i [15,16,17,18,19,20,21,22,23,24,25,26,27,28,29,30,31,32,33,34,35,36,37,38,39,40,41,42].

The risk of bias in the included non-randomized studies was assessed using the ROBINS-I (Risk Of Bias In Non-randomized Studies of Interventions) tool. This instrument evaluates seven domains of bias, including confounding, selection of participants, classification of interventions, deviations from intended interventions, missing data, measurement of outcomes, and selection of the reported result. Each study was independently assessed by two reviewers, and discrepancies were resolved through discussion. Most studies showed a low to moderate overall risk of bias. However, a few studies presented a serious risk of bias, particularly due to confounding factors and limitations inherent to retrospective designs (Figure 2a,b, Supplementary Table S1).

## 3. Results

Our initial search yielded 2162 studies, which were reduced to 2123 unique records after removing duplicates. Through a rigorous screening process involving title, abstract, and full-text reviews, we identified 28 studies that met our predefined eligibility criteria for inclusion in this systematic review (Table 1).

### 3.1. Features of Included Studies

We included studies investigating the role of both RARC and ORC, with a particular focus on their impacts on sexual function. Additionally, we explored the role of nerve-sparing surgery, encompassing both organ-sparing and nerve-sparing techniques. With the advent of robotic surgery and the proliferation of various robotic platforms, we opted not to conduct a platform-specific analysis. Instead, our focus was on studies examining the preservation of sexual function regardless of the surgical approach, and we also included studies investigating the impact of adjuvant therapies, such as radiotherapy (Table 2).

### 3.2. Assessment of Sexual Function in Female Patients

**Figure 3:**
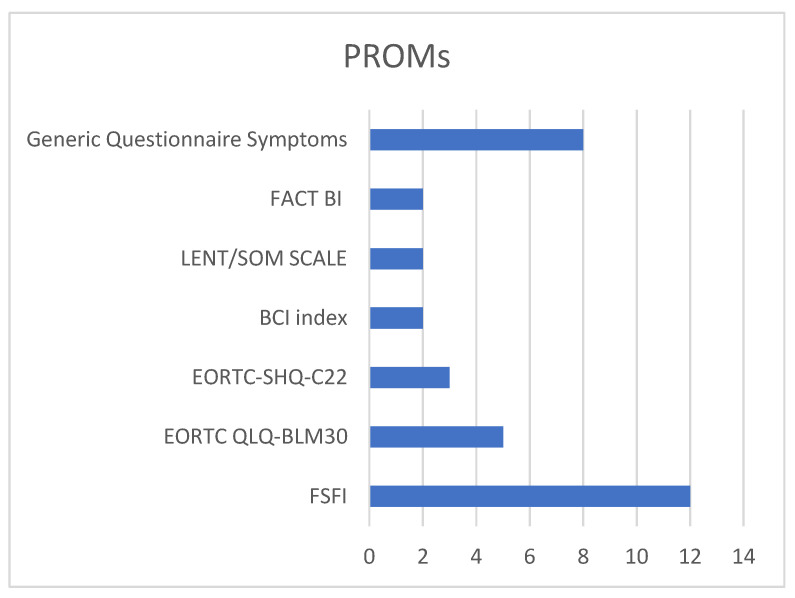
Type of questionnaire used in the studies.

In our screened articles, there was a wide variety of screening instruments employed, highlighting a lack of consensus on the most suitable questionnaires for assessing female patients. This diversity stemmed from the numerous aspects of female sexual health that require exploration. Our review identified several questionnaires, including the European Organization for Research and Treatment of Cancer (EORTC) QLQ-C22 and the EORTC FACT-BL, the Bladder Cancer Index (BCI), the LENT SOMA, and the Female Sexual Function Index (FSFI) [43]. Among the studies we examined, seven utilized generic questionnaires focusing on symptoms during sexual intercourse, while one study employed a more specialized questionnaire addressing sexual desire and symptoms. The EORTC QLQ-C30 and its bladder-cancer-specific module, FACT-BL, are general cancer quality of life tools that include limited assessment of sexual health and do not contain items specific to female patients. The BCI is a validated, disease-specific instrument that evaluates urinary, bowel, and sexual domains, but again, its sexual function section is not gender-specific. The LENT-SOMA questionnaire is designed to evaluate long-term treatment-related symptoms, including sexual side effects, but it remains broad and not tailored to female patients. In contrast, the Female Sexual Function Index (FSFI) is a validated, multidimensional tool specifically developed to assess female sexual function, covering domains such as desire, arousal, lubrication, orgasm, satisfaction, and pain. Among these instruments, the FSFI is the only one explicitly designed to capture the complexity of female sexual health, making it particularly relevant in evaluating outcomes in women after radical treatment for bladder cancer [15,16,17,18,19,20,21,22,23,24,25] (Figure 3).

### 3.3. Timing of Questionnaire and Sexual Activity

Three studies administered questionnaires both pre- and postoperatively, providing detailed results [17,19,23]. Of the 18 studies that included data on sexual activity before and after cystectomy, a median of 23 female patients across these studies reported being sexually active prior to surgery, while a median of 11 female patients reported being sexually active post-surgery. Additionally, some studies reported on the time to recovery of sexual function after surgery, with most patients regaining sexual function within 6 months.

### 3.4. Radical Cystectomy in Female Patients

The majority of the articles explored the impact of RC on female sexuality. Of the 29 articles, 11 investigated the role of ORC and evaluated the differences between various types of urinary diversion [17,20,22,23,26,27,28,29,30,31,32]. Three studies specifically compared the outcomes of IC, ON, and IP [17,32,33]. Another three studies directly compared a robotic approach to ORC [22,27,31]. Two other studies discovered the impact of RC on oncological outcomes without dividing by type of surgery [34,35].

### 3.5. Robot-Assisted Radical Cystectomy (RARC)

The role of minimally invasive surgery has significantly advanced the field of urology in recent decades. With the proliferation of various robotic platforms, understanding their impacts on the sexual function of patients undergoing RC is crucial. Eight studies in our review assessed sexual function after RC, demonstrating a trend towards the increasing use of nerve-sparing techniques and ON. These advancements may be attributed to the ergonomic features of robotic surgery, as exemplified by the growing adoption of robot-assisted radical prostatectomy. One study included the largest number of patients but lacked a detailed analysis of different diversion types [16,21,22,27,31,36,37,38].

### 3.6. Organ-Sparing Radical Cystectomy

Regarding this aspect, nine studies explored the impact of organ-sparing surgery on female patients, specifically focusing on reproductive organ-sparing surgery and vaginal-sparing surgery [17,18,21,26,28,29,36,38,39]. Six of them also included vaginal-sparing surgery [22,30,33,37]. The results consistently demonstrated that organ-sparing surgery improved the perception of sexual intercourse. Notably, this trend was particularly evident in studies describing robotic surgery procedures performed on younger patients.

### 3.7. Type of Urinary Diversion

From the perspective of urinary diversion used during radical cystectomy, thirteen studies analyzed different types of diversion, particularly focusing on neobladder and ileal conduit [17,22,30,31,32,33,37]. Eight studies compared the two techniques using both open and robotic approaches, although they did not specify whether the reconstructive phase was performed intracorporeally or extracorporeally. In contrast, Indiana pouch and ureterocutaneostomy were less frequently reported, typically involving older patients or those with more advanced disease stages.

### 3.8. Radiation Therapy for Radical Cystectomy

While several studies included an assessment of radiotherapy and its impact on quality of life, few delved into the specific role of radiotherapy as either neoadjuvant or adjuvant therapy for bladder cancer (BC). Moreover, many studies did not differentiate between male and female sexual function, leading us to exclude these articles from our analysis. The specific impact of radiotherapy on female sexual function was addressed in only a limited number of studies. These studies primarily employed the FACT-B and EORTC QLQ-C30 questionnaires and generally reported a decrease in sexual desire [40,41,42].

## 4. Discussion

Careful patient selection is crucial to ensure that cancer control is not compromised. Factors to consider include age, menopausal status, sexual function, fertility goals, and gynecologic history. Women with good overall health, younger age (though not a strict requirement), and a desire for organ preservation and neobladder reconstruction are generally considered better candidates for organ-sparing cystectomy [13,17,36].

Consequently, addressing sexual dysfunction in BC patients necessitates a multifaceted and comprehensive approach.

Our initial review aimed to comprehensively understand the role of functional outcomes, particularly sexual function, in patients with MIBC undergoing radical therapy or radiotherapy. Despite the development of numerous robotic platforms that have enhanced surgical precision and technological advancements in radiotherapy, the aspect of sexuality after treatment continues to receive limited attention. The various articles we analyzed did not exhibit a consistent use of questionnaires related to sexuality, with diverse questionnaires being employed, and a lack of standardization among them.

One of the primary issues we encountered was the lack of interest from the scientific community regarding female sexuality. In the past two decades, the scientific community has focused extensively on male sexuality following radical prostatectomy, and companies have heavily invested in devices aimed at improving any potential deficits. In the realm of female sexuality, we observed a tendency to use generic questionnaires that do not comprehensively explore all aspects of sexual comfort. Our analysis revealed that the EORTC BLM 30 is the only questionnaire that adequately addresses female sexuality, covering topics such as orgasm, difficulty with penetration, vaginal discomfort, and inadequate lubrication.

According to EAU guidelines, the treatment for MIBC involves either a bladder-sparing treatment or a radical treatment that includes removal of the bladder, the anterior wall of the vagina, and the annexes. As we can infer from various articles, the role of organ-sparing surgery is a feasible technique, especially for women who wish to maintain a good quality of life.

In 2004, Craig et al. reported in their study the experience of 27 women who underwent ORC with either ON or IC and found that the type of urinary diversion did not impact sexual function, but the type of organ-sparing surgery performed, particularly the vaginal- and nerve-sparing techniques, played a significant role [17].

Referring again to an open approach, in a 2016 qualitative study, Gupta et al. described the impact of vaginal-sparing surgery in 13 women, stating that the impact was not only in terms of difficulty during sexual intercourse, but also in terms of psychological barriers and body image, and underlined the need for adequate preoperative counseling [26].

Regarding quality of life after RC, the comparison between ON and orthotopic surgical reconstruction has been explored in various studies. In 2011, Badawy et al. [39] noted that in a cohort of 78 women who underwent RC and ON, while organ-sparing surgery was performed, a specific questionnaire on sexual function was not applied. However, it was evident that 20% of patients had a decrease in sexual desire and 23% experienced difficulties in reaching orgasm, regardless of the preservation of sexual organs.

Later, in 2015, Roshdy et al. [28] analyzed the impact of the neobladder in 24 patients with ON, demonstrating, unlike Craig et al. [17], that this technique guarantees optimal functional outcomes, ensuring a good overall sexual satisfaction for the majority of patients (91%), who regained sexual activity within 6 months.

Wishahi et al. and Tuderti et al. [21,29] analyzed a similar number of patients who underwent RC and sexual organ sparing with two different approaches, open surgery and robotic surgery, respectively. At the end of the follow-up, both studies demonstrated how the integrity of the uterosacral ligaments is fundamental for the recovery of continence and sexual health, independently of the surgical type. Determining factors seemed to be the pathological characteristics of the lesion and the patient’s age.

The type of urinary diversion does not appear to significantly impact quality of life in terms of sexual function, as the administered questionnaires did not reveal substantial differences. In our retrospective study conducted on a cohort of patients who underwent radical cystectomy with either Padua ileal neobladder or ileal conduit, no significant differences were observed. Patients who received the neobladder reported higher scores in body image perception, but not in functional outcomes [10].

Susanna et al. [19], in a cohort of 73 patients, demonstrated (with the limitations of their study) that there is no significant advantage in terms of improvement in quality of life between ON and IC.

More recently, there have been several studies that have addressed the issue of robotic surgery. Lavalee et al. [38], in particular, in their study, analyzed 23 patients who underwent RARC with organ-sparing surgery. This study has shown that organ-sparing surgery can be a safe technique in terms of oncological outcomes and can be performed in sexually active women with solitary muscle-invasive tumors that do not involve the trigone and are organ-confined. A limitation of this study is that sexual function is analyzed only through questionnaires related to the resumption of functional activity after surgery and questionnaires related to sexual discomfort are not applied.

In 2023, Cisternino et al. [27] described their technique for preserving female genital organs in patients undergoing RC. In their cohort of 14 patients who underwent radical treatment with ON, the preservation of reproductive organs correlated with significant improvement not only in the sexual aspect but also in the psycho-emotional aspect. Moreover, in selected patients, good results could also be obtained in terms of fertility.

Other studies analyzed the impact of vaginal preservation on sexuality. In particular, Clement et al. [22,30] found that vaginal preservation, often associated with continent diversions, did not significantly improve functional outcomes, but at 12 months, the results of the EORTC-QLQ-BLM30 questionnaire showed an improvement compared to 6 months post-operative, with a trend of worsening of 13% compared to baseline.

In 2024, Pacchetti et al. [37] analyzed 22 patients who underwent RARC, of which, 19 underwent a vaginal-sparing technique, supporting the reproducibility of the technique and the achievement of functional outcomes, mainly in young patients after neoadjuvant therapy (NAC) and an adequate patient selection [44].

In a single study, the EORTC SHQ-C22 questionnaire was analyzed. In their cross-sectional study of 104 patients who underwent RARC and were administered the questionnaire, Milling et al. [16] found that 43% of patients in this case did not achieve orgasm and 82% showed dyspareunia. Sexual discomfort and relative discomfort with modifications to body image and also a sense of vaginal congestion were cited as radical impacts that could negatively affect sexuality [44].

Lind et al. [31], in their cohort of 40 patients with a prevalence of non-continent urinary diversion, analyzed sexual satisfaction with the BCI questionnaire and observed that 31% of patients demonstrated that they were very satisfied. In this study, they underlined the importance of post-operative counseling to encourage sexual intercourse and how this can have a significant impact also on social well-being.

In patients undergoing RT, the mean age is higher, in accordance with guidelines, and radiation therapy is used both as palliative therapy and in trimodal treatment regimens [45]. The included studies do not address issues related to functional outcomes in terms of sexuality. Given that the population is generally older, it is difficult to find an objective way to compare groups. Despite these difficulties, Allareddy et al. [40], without distinguishing between sexes (male vs. female), showed that only 27% of the population undergoing radiation therapy was interested in sexual function. Mak et al. [42], in their study of 14 patients undergoing TMT, found a higher quality of life compared to patients undergoing RC. Considering the results obtained in the EORTC-QLQ-BLM30 questionnaire in their study, they analyzed how the impact of radiotherapy negatively affected quality of life and sexuality, with a result on the LENT/SOM scale of four in 57% of cases.

Pelvic irradiation may lead to vaginal dryness, fibrosis, and dyspareunia, contributing to female sexual dysfunction and urological conditions [46]. However, the sexual health outcomes in this population remain underreported, partly due to the advanced age of many patients undergoing radiotherapy, who are less frequently included in sexual function assessments. This gap highlights the need for more inclusive and age-sensitive research to fully understand the implications of radiotherapy on sexual quality of life [47].

Regarding the heterogeneity of questionnaires, the FSFI and the EORTC QLQ-BLM30 are among the most commonly used instruments to assess sexual function in women following radical cystectomy. The FSFI is a validated, multidimensional questionnaire specifically designed to evaluate key domains of female sexual function, including desire, arousal, lubrication, orgasm, satisfaction, and pain. It offers a comprehensive overview but may lack specificity for bladder-cancer-related issues. In contrast, the EORTC QLQ-BLM30 is a bladder-cancer-specific module developed by the European Organization for Research and Treatment of Cancer, which complements the core QLQ-C30 questionnaire [48]. Although it includes items related to body image and sexual functioning, its coverage of female-specific sexual concerns remains limited. The combined use of these tools can provide valuable insights but also highlights the need for more tailored, disease-specific instruments for this patient population.

This review had a broad scope, including a wide range of studies. However, studies specifically examining sexual function in bladder cancer were limited, and those focusing on female sexual function were even rarer. Most studies investigated unmet needs or overall quality of life, with sexual function considered within this broader context. Therefore, the findings might be influenced by this broader perspective. Many of the included studies were conducted more than a decade ago. While this review encompassed all BC treatment methods, cystectomy has unique anatomical consequences that significantly impact sexual function. Therefore, it is crucial to differentiate the results of cystectomy studies from those involving other treatments. A further limitation is the scarcity of studies evaluating quality of life in terms of sexuality in patients undergoing radiotherapy. This represents a major gap in the literature, as radiotherapy may have distinct physical and psychological effects on female sexual function that remain poorly understood and underexplored. Addressing this lack of data is essential to ensure a more comprehensive understanding of the impact of all radical treatment modalities.

## 5. Conclusions

This systematic review highlights the significant prevalence and impact of FSD following radical treatment for MIBC, with poor evidence on radiotherapy. While organ-sparing and nerve-sparing surgical techniques show promise, high-quality and standardized studies are lacking. Further research should prioritize patient-centered outcome measures and include qualitative insights to guide individualized therapeutic decisions.

## Figures and Tables

**Table 1:** Description of study with demographic features, we considered both median and average in the statistical report.

Study	Study Design	Center Involved	Female Eligible Patients	Age (Median or Average)	Follow-Up	Pre RC Sexual Activity	Intervention
Craig et al., 2004 [17]	Retrospective	Single center	34	m54.79	24.2 (15–65)	29	RC
Gupta et al., 2020 [26]	Retrospective	Single center	16	67.5 (60.5–79.5)	12	4	RC
Tyritzis et al., 2013 [36]	Retrospective	Single center	8	N/A	N/A	6	RC
Ali el dein et al., 2013 [18]	Retrospective	Single center	15	m42	70 (37–99)	N/A	RC
Siracusano et al., 2018 [35]	Prospective	Multicentric	73	67 (44–86) ON 67 (44–86) IC	43 (16–138) ON 54 (6–153) IC	N/A	RC
Pacchetti et al., 2024 [37]	Retrospective	Multicentric	22	62 (57–66)	29 (16–44)	15	RC
Lavallèè et al., 2021 [38]	Retrospective	Multicentric	23	54 (34–79)	20 (7–151)	23	RC
Milling et al., 2024 [16]	Retrospective	N/A	289	72 (65–75)	N/A	117	RC
Cisternino et al., 2023 [27]	Retrospective	Single center	14	57.6 (30–65)	N/A	N/A	RC
Wenk et al., 2013 [33]	Retrospective	Single center	322	m65.89	N/A	N/A	RC
Gacci et al., 2013 [34]	Retrospective	Multicentric	41	m67.3	60.1 (36–122)	N/A	RC
Kretschmer et al., 2016 [24]	Retrospective	Single center	19	N/A	m48	N/A	RC
Roshdy et al., 2015 [28]	Retrospective	Single center	70	51 (45–60)	48 (36–58)	24	RC
Wishahi et al., 2015 [29]	Retrospective	Single center	30	m39.4	N/A	13	RC
Tuderti et al., 2020 [21]	Retrospective	Single center	11	m47.1	28 (14–51)	11	RC
Badawy et al., 2021 [39]	Retrospective	Single center	78	*42.4 (36–58)*	m62	78	RC
Clements et al., 2022 [30]	Prospective	Single center	88	N/A	m24	N/A	RC
Clements et al., 2023 [22]	Retrospective	Single center	15	N/A	m24	N/A	RC
Lind et al., 2023 [31]	Prospective	Multicentric	40	69.5 (46–87)	12	40	RC
Philips et al., 2023 [25]	Prospective	Single center	85	N/A	60	25	RC
Henningsohn et al., 2003 [32]	Retrospective	Multicentric	91	*m65.0*	N/A	N/A	RC
Fodkal et al., 2004 [41]	Retrospective	Single center	16	N/A	29 (18–102)	7	RT
Mak et al., 2006 [42]	Prospective	Single center	41	66 (59–71)	5.6	29	RT
Volkmer et al., 2004 [23]	Retrospective	Single center	86	61	12	17	RC
El-bahnasawy et al., 2011 [19]	Retrospective	Single center	73	*52.3 ± 6.5*	N/A	73	RC
Both et al., 2014 [20]	Retrospective	Single center	71	67 (39–91)	N/A	41	RC
Nordstrom et al., 1992 [15]	Retrospective	Single center	26	N/A	N/A	6	RC
Allareddy et al., 2006 [40]	Retrospective	Single center	58	64.4	N/A	N/A	RT

**Table 2:** Description of study with type of surgical technique and pT stage.

Study	Type of Surgery	Type of Urinary Diversion	Organ Sparing	Vaginal Sparing	Pathological Stage (Number of Patients)
Craig et al., 2004 [17]	ORC	IC, ON, IP	N/A	10	T1: 20 T2: 14
Gupta et al., 2020 [26]	ORC	IC and ON	N/A	13	T1: 8 T2-T4: 8
Tyritzis et al., 2013 [36]	RARC	ON	8	N/A	N/A
Ali el dein et al., 2013 [18]	N/A	ON	13	N/A	T2: 14 T4: 1
Siracusano et al., 2018 [35]	N/A	IC and ON	N/A	N/A	T1-Cis: 21 T2: 23 T3: 29
Pacchetti et al., 2024 [37]	RARC	ON	N/A	19	T1-Cis: 13 T2: 3 T3: 6
Lavallèè et al., 2021 [38]	RARC	IC and ON	23	N/A	T1-Cis: 10 T2: 13
Milling et al., 2024 [16]	RARC	IC and ON	N/A	N/A	T2-T4: 86
Cisternino et al., 2023 [27]	RARC and ORC	ON	N/A	N/A	T1-Cis: 8 T2: 4 T3: 2
Wenk et al., 2013 [33]	N/A	IC, ON, UCS, IP	N/A	6	T1-Cis: 19 T2: 16
Gacci et al., 2013 [34]	N/A	IC, ON, UCS	N/A	N/A	N/A
Kretschmer et al., 2016 [24]	N/A	N/A	N/A	N/A	N/A
Roshdy et al., 2015 [28]	ORC	ON	24	N/A	T1-Cis: 4 T2: 20
Wishahi et al., 2015 [29]	ORC	ON	13	N/A	T1-Cis: 4 T2: 6 T3: 3
Tuderti et al., 2020 [21]	RARC	ON	11	N/A	T1-Cis: 7 T3: 4
Badawy et al., 2021 [39]	N/A	ON	9	N/A	N/A
Clements et al., 2022 [30]	RARC and ORC	IC and ON	N/A	49	N/A
Clements et al., 2023 [22]	RARC and ORC	IC and ON	N/A	10	N/A
Lind et al., 2023 [31]	RARC and ORC	IC, ON, UCS	N/A	N/A	T1-Cis: 12 T2: 22 T3: 5 T4: 1
Philips et al., 2023 [25]	N/A	N/A	N/A	N/A	N/A
Henningsohn et al., 2003 [32]	ORC	IC, ON, IP	N/A	N/A	N/A
Volkmer et al., 2004 [23]	ORC	ON	N/A	N/A	N/A
El-bahnasawy et al., 2011 [19]	ORC	IC, ON	N/A	N/A	N/A
Both et al., 2014 [20]	ORC	IC, ON, IP	N/A	N/A	N/A
Nordstrom et al., 1992 [15]	N/A	N/A	N/A	N/A	N/A

## Data Availability

Our data are available on PROSPERO platform.

## Supplementary Materials

The following supporting information can be downloaded at: https://www.mdpi.com/article/10.3390/jpm15090415/s1. Supplementary Materials: PRISMA_2020_checklist, Reference [49] is cited in the supplementary materials; Table S1: Risk of bias assessment using the ROBINS-I tool for all included non-randomized studies.

## Abbreviations

The following abbreviations are used in this manuscript:

BC	Bladder cancer
MIBC	Muscle-invasive bladder cancer
NMIBC	Non-muscle-invasive bladder cancer
QoL	Quality of life
FSD	Female sexual dysfunction
