# Supplementary material for: Female Sexual Function After Radical Treatment for MIBC: A Systematic Review
Source: J Pers Med. 2025 Sep 2;15(9):415. doi: 10.3390/jpm15090415 (PMC12471525; doi:10.3390/jpm15090415)
Supplement: Supplementary file 1 [file jpm-15-00415-s001.zip › jpm-3758643-supplementary.pdf]

Studio	Confounding	Selection	Classification	Deviation from intervention	Missing data	Measurement of outcome	Selection of reported result	Overall Bias
Craig et al., 2004 ([17])	Moderate	Low	Low	Low	Moderate	Moderate	Moderate	Low
Gupta et al., 2020 ([26])	Moderate	Low	Moderate	Low	Low	Low	Moderate	Moderate
Tyritzis et al., 2013 ([36])	Critical	Low	Low	Low	Low	Low	Low	Low
Ali el dein et al., 2013([18])	Critical	Low	Low	Low	Low	Low	Low	Low
Siracusano et al., 2018 ([35])	Critical	Low	Low	Low	Low	Moderate	Low	Low
Pacchetti et al., 2024 ([37])	Critical	Low	Low	Low	Low	Moderate	Low	Low
Lavallèè et al., 2021 ([38])	Critical	Low	Low	Low	Low	Moderate	Low	Low
Milling et al., 2024([16])	Critical	Low	Moderate	Low	Low	Moderate	Low	Moderate
Cisternino et al., 2023 ([27])	Critical	Low	Moderate	Moderate	Low	Low	Low	Moderate
Wenk et al., 2013 ([33])	Critical	Low	Moderate	Moderate	Low	Low	Low	Moderate
Gacci et al., 2013 ([34])	Moderate	Moderate	Moderate	Moderate	Moderate	Moderate	Moderate	Moderate
Kretschmer et al., 2016 ([24])	Critical	Critical	Low	Low	Low	Moderate	Moderate	Moderate
Roshdy et al., 2015 ([28])	Moderate	Critical	Low	Low	Low	Moderate	Moderate	Moderate
Wishahi et al., 2015 ([29])	Moderate	Moderate	Moderate	Moderate	Moderate	Moderate	Moderate	Moderate
Tuderti et al., 2020 ([21])	Low	Low	Low	Low	Moderate	Moderate	Moderate	Low
Badawy et al., 2021 ([39])	Critical	Moderate	Moderate	Moderate	Critical	Moderate	Moderate	Low
Clements et al., 2022 ([30])	Moderate	Low	Low	Moderate	Low	Moderate	Low	Low
Clements et al., 2023 ([22])	Low	Low	Low	Moderate	Low	Moderate	Low	Low
Lind et al., 2023 ([31])	Moderate	Low	Moderate	Moderate	Low	Critical	Critical	Low
Philips et al., 2023 ([25])	Critical	Low	Critical	Moderate	Low	Moderate	Low	Low
Henningsohn et al., 2003 ([32])	Moderate	Moderate	Critical	Moderate	Critical	Critical	Critical	Low

Fodkal et al., 2004 ([41])	Moderate	Moderate	Critical	Moderate	Critical	Critical	Critical	Low
Mak et al., 2006 ([42])	Moderate	Moderate	Critical	Moderate	Critical	Moderate	Moderate	Low
Volkmer et al., 2004 ([23])	Moderate	Critical	Moderate	Critical	Critical	Critical	Critical	Low
El-bahnasawy et al., 2011 ([19])	Moderate	Critical	Moderate	Critical	Critical	Critical	Critical	Low
Both et al., 2014 ([20])	Moderate	Critical	Moderate	Critical	Critical	Critical	Critical	Low
Nordstrom et al., 1992 ([15])	Moderate	Critical	Moderate	Critical	Critical	Critical	Critical	Low

Supplementary Table S1. Risk of bias assessment using the ROBINS-I tool for all included non-randomized studies. The evaluation covers seven bias domains: confounding, selection of participants, classification of interventions, deviations from intended interventions, missing data, measurement of outcomes, and selection of the reported result. Each domain was rated as low, moderate, serious, critical risk of bias, or no information. The overall risk of bias reflects the highest level of bias observed across any domain.
